# Impact of the COVID‐19 pandemic on the time to emergency endoscopy and clinical outcomes in patients with upper gastrointestinal bleeding

**DOI:** 10.1002/deo2.310

**Published:** 2023-11-10

**Authors:** Takumi Komatsu, Yoshinori Sato, Yuichiro Kuroki, Yoshihito Yoshida, Natsumi Aoyama, Yoshihiko Iijima, Yusuke Nakamoto, Masaki Kato, Hirofumi Kiyokawa, Kenichiro Tanabe, Koutaro Matsunaga, Tadateru Maehata, Hiroshi Yasuda, Nobuyuki Matsumoto, Keisuke Tateishi

**Affiliations:** ^1^ Department of Gastroenterology St Marianna University School of Medicine Kanagawa Japan; ^2^ Department of Gastroenterology St Marianna University School of Medicine, Yokohama Seibu Hospital Kanagawa Japan; ^3^ Department of Gastroenterology Kawasaki Municipal Tama Hospital Kanagawa Japan; ^4^ Pathophysiology and Bioregulation St. Marianna University Graduate School of Medicine Kanagawa Japan

**Keywords:** COVID‐19, emergencies, endoscopy, gastrointestinal hemorrhage, retrospective studies

## Abstract

**Objectives:**

To investigate endoscopic management and clinical outcomes in patients with non‐variceal upper gastrointestinal (GI) bleeding during the coronavirus disease 2019 pandemic.

**Methods:**

We retrospectively analyzed the data of 332 patients with non‐variceal upper GI bleeding who underwent emergency upper GI endoscopy at three hospitals during the pandemic (April 2020–June 2021) and before the pandemic (January 2019–March 2020). The number of emergency upper GI endoscopies, time from hospital arrival to endoscopy, mortality within 30 days, rebleeding within 30 days, interventional radiology (IVR)/surgery requirement, composite outcome, rates of endoscopic hemostasis procedures, and second‐look endoscopy were investigated using logistic regression.

**Results:**

Overall, 152 and 180 patients underwent emergency upper GI endoscopies during and before the pandemic, respectively. The mean time from arrival to endoscopy was longer during the pandemic than before it (11.7 vs. 6.1 h, *p <* 0.01). Multivariate analysis revealed that mortality within 30 days (odds ratio [OR]: 2.27, *p* = 0.26), rebleeding within 30 days (OR: 0.43, *p* = 0.17), IVR/surgery requirement (OR: 1.79, *p* = 0.33), and composite outcome (OR: 0.98, *p* = 0.96) did not differ significantly between the periods; conversely, endoscopic hemostasis procedures (OR: 0.38, *p* < 0.01) and second‐look endoscopies (OR: 0.04, *p* < 0.01) were less likely to be performed during the pandemic than before it.

**Conclusions:**

Although the time from arrival to endoscopy was significantly longer during the pandemic, it did not affect mortality and rebleeding.

## INTRODUCTION

Three years have passed since coronavirus disease 2019 (COVID‐19) was declared a pandemic. COVID‐19 is a respiratory infection caused by the novel coronavirus, severe acute respiratory syndrome coronavirus, which is transmitted via aerosols.^1^, ^2^ Accordingly, the pandemic has massively impacted all types of medical care procedures that entail a high risk of droplet dispersal and aerosol generation,^3^ such as upper gastrointestinal (GI) endoscopies. Therefore, recommendations suggest avoiding unnecessary endoscopies during pandemics.^4^, ^5^ Furthermore, adequate infection control measures are important when conducting endoscopies.

Non‐variceal upper GI bleeding (NVUGIB) is a common medical emergency that requires emergency endoscopy; its mortality rate is reported to be 2.5%–7.8%.^6^, ^7^ Thus, it must be conducted rapidly after the patient arrives at the hospital. However, during the COVID‐19 pandemic, the number of emergency upper GI endoscopies performed reportedly decreased by 30%–40%.^8^ The potential effect of this on the mortality of patients with NVUGIB is a matter of concern. However, only limited studies have investigated the endoscopic management and clinical outcomes of these patients during the COVID‐19 pandemic.^9^, ^10^, ^11^ Moreover, aspects of endoscopic management that may influence the risks of mortality and rebleeding, such as the time from arrival to emergency endoscopy, have not been fully investigated.

Therefore, we conducted this three centers study on the endoscopic management and clinical outcomes of patients with NVUGIB during the COVID‐19 pandemic in Japan.

## METHODS

### Patient selection

**FIGURE 1:**
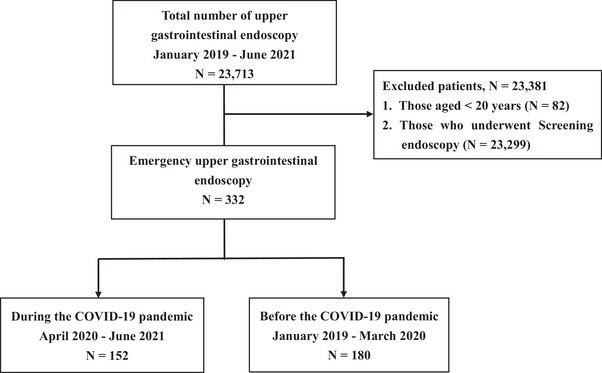
Flowchart of study participants.

This retrospective cohort study was conducted at three tertiary centers in Japan. The study participants comprised outpatients aged ≥20 years who presented with hematemesis or tarry stool, were diagnosed with NVUGIB, and were urgently hospitalized between January 2019 and June 2021. During the study period, 23,713 patients underwent upper GI endoscopies; among them, those aged ≤20 years (*n* = 82) and those who underwent screening endoscopy (*n* = 23,299) were excluded. Thus, 332 hospitalized patients who underwent emergency upper GI endoscopy were included in the study (Figure 1). Based on when the first COVID‐19 State of Emergency was announced in Japan (i.e., at the start of April 2020), the study period was divided into two parts: April 2020–June 2021 (during the COVID‐19 pandemic) and January 2019–March 2020 (before the COVID‐19 pandemic; Figure 1).

### Data collection

Data were collected from electronic medical records and the hospitals’ endoscopy databases. The collected baseline characteristics were sex, age, underlying diseases, and medications used. Antiplatelet users were defined as users of aspirin, thienopyridine, and cilostazol. Anticoagulant users were defined as users of warfarin and direct oral anticoagulants. We evaluated the vital signs on arrival as well as the shock index (≥1.0; heart rate divided by the systolic blood pressure)^12^; furthermore, the Glasgow–Blatchford score (GBS),^13^ AIMS65 score,^14^ and Rockall score^15^ were evaluated as severity indices. We further evaluated the management on arrival at the hospital. We also recorded the number of patients who received intravenous proton pump inhibitor (PPI) infusion (omeprazole at 40 mg/day) before upper GI endoscopy. Regarding in‐hospital management, we evaluated the total units of blood transfused. As COVID‐19‐related factors, we evaluated chest computed tomography (CT), COVID‐19 antigen test, and polymerase chain reaction (PCR) test findings. A positive COVID‐19 test was defined as a positive result from either genetic testing (PCR or loop‐mediated isothermal amplification) or antigen testing (quantitative) using samples such as saliva or pharyngeal/nasal swabs.

### Endoscopic management

Emergency upper GI endoscopy was performed by one of 19 hospital‐affiliated endoscopists, with trainees supervised by an expert with at least 10 years of experience with endoscopy. Standard precautions before the COVID‐19 pandemic involved the use of gloves, unwoven masks, and gowns. During the COVID‐19 pandemic, endoscopists used full personal protective equipment (PPE), including gloves, N95 masks, eye goggles or a face shield, and gowns.^16^ Furthermore, during the COVID‐19 pandemic, all patients wore droplet‐preventing masks during endoscopy, as these prevent aerosol transmission.^17^ Patients who had hematemesis, melena, or both,^7^ or hemorrhagic shock, or requiring transfusion were indicated for urgent endoscopy. Regarding the time of endoscopy, the time to emergency upper GI endoscopy, time of the day when endoscopy was performed, procedural time were evaluated. The time to emergency upper GI endoscopy was defined as the time from hospital arrival to the start of scope insertion. We defined “Urgent endoscopy” as an endoscopy performed within 6 h of the patient's arrival, and “Early endoscopy” as an endoscopy performed within 6–24 h after patient's arrival.^7^ The time of the day when the endoscopy was performed was classified as either being within regular working hours (9:00–17:00 h) or outside regular working hours (17:01–8:59 h).

Next, the diagnosis, bleeding site, Forrest classification,^18^ and endoscopic hemostasis were evaluated. The endoscopes used were GIF‐Q260J or GIF H290‐T (Olympus); both were equipped with water jets. Regarding the endoscopic diagnosis, “others” comprised erosion, acute gastric mucosal lesion, portal hypertensive gastropathy, or hyperplastic polyps. Cases in which the bleeding source could not be endoscopically identified were classed as “unidentified.” The bleeding site was classified as one of the following seven sites: the esophagus, esophagogastric junction, stomach (parts: upper [U], middle [M], or lower [L]),^19^ duodenal bulb, and the second part of the duodenum. For endoscopic hemostasis, high‐frequency coagulation with coagulation forceps (Coagrasper, Olympus) or hemoclips (HX‐610‐135, Olympus) were generally used to treat peptic ulcer and Mallory–Weiss syndrome.^20^, ^21^, ^22^ Argon plasma coagulation was used for malignant neoplasm and gastric antral vascular ectasia.^23^ These hemostasis methods were set as the basis for treatment. Interventional radiology (IVR) or surgery was opted for in case of difficulties with achieving endoscopic hemostasis.

### Clinical outcomes

The primary outcomes were mortality within 30 days, rebleeding within 30 days, need for IVR/surgery, and composite outcome comprising all three.^24^ The secondary outcomes were time to emergency upper GI endoscopy, rate of endoscopic hemostasis, rate of second‐look endoscopy, need for blood transfusion, and prolonged hospitalization (≥14 days). In a subgroup analysis, we also evaluated these outcomes in a high‐risk group of patients with a GBS ≥12 points.^25^ Next, we evaluated the rates of endoscopic hemostasis in the groups receiving and not receiving omeprazole before endoscopy. Rebleeding was defined by the presence of tarry stool, decreased hemoglobin levels (≥2 g/dL), or hemodynamic changes (heart rate ≥110 bpm; systolic blood pressure ≤90 mmHg) after hemostasis at the first endoscopy.^12^ The cause of death was determined based on findings from blood tests, multiple imaging modalities, or autopsies.

### Statistical analyses

Continuous variables are expressed as averages. In univariate analyses, the *χ* ^2^ test and Fisher's exact test were used to compare categorical variables, whereas Student's *t*‐test was used to compare continuous variables. A logistic regression analysis was performed to calculate the odds ratios (ORs) with a 95% confidence interval of the risks for clinical outcomes. Multivariate analyses were adjusted for sex, an AIMS65 score ≥2,^14^ and a complete Rockall score ≥5^15^ because these are associated with rebleeding and mortality in patients with upper GI bleeding. All statistical analyses were performed using STATA version 16 (StataCorp) and *p* < 0.05 was considered significant.

## RESULTS

### Patient characteristics

The study analyzed 332 patients (228 men and 104 women), with a mean age of 72.5 ± 15.6 years. Their baseline characteristics are summarized in Table 1. Emergency upper GI endoscopies were performed in 180 and 152 patients before and during the COVID‐19 pandemic, respectively. Of the 10,116 and 13,597 upper GI endoscopy procedures conducted during and before the COVID‐19 pandemic, 87 (0.9%) and 139 (1.0%) were urgent cases, respectively (*p* = 0.20), showing no significant difference. Regarding comorbidities, chronic kidney disease (*p* = 0.04) and diabetes (*p* = 0.03) were significantly higher during the COVID‐19 pandemic than before. Regarding blood tests, the mean hemoglobin levels were significantly lower during the COVID‐19 pandemic than before (8.4 g/dL vs. 9.0 g/dL; *p* = 0.048), and the mean PT‐INR levels were significantly higher during the COVID‐19 pandemic than before (1.4 vs. 1.1; *p* = 0.01). Severity of upper GI bleeding, and management at hospital arrival did not differ between the two periods.

**TABLE 1:** Clinical characteristics of the patients.

Characteristics	During the COVID‐19 pandemic (*n* = 152)	Before the COVID‐19 pandemic (*n* = 180)	*p*‐value
Sex (male/female), *n* (%)	114 (75.0)/38 (25.0)	114 (63.3)/66 (36.7)	0.022
Age (mean ± SD), years	72.8 ± 15.7	72.3 ± 15.4	0.789
**Comorbidities, *n* (%)**			
Cardiovascular disease	45 (29.6)	42 (23.3)	0.195
Cerebrovascular disease	18 (11.8)	18 (10.0)	0.591
Chronic kidney disease	30 (19.7)	21 (11.7)	0.042
Hepatic disease	16 (10.5)	17 (9.4)	0.743
Diabetes	35 (23.0)	25 (13.9)	0.031
Respiratory disease	2 (1.3)	1 (0.6)	0.466
Malignant neoplasms	27 (17.8)	24 (13.3)	0.265
**Medication, *n* (%)**			
Antiplatelet drugs	33 (21.7)	28 (15.6)	0.150
Anticoagulant drugs	29 (19.1)	24 (13.3)	0.154
DAPT	3 (2.0)	3 (1.7)	0.834
NSAIDs	26 (17.1)	25 (13.9)	0.418
Proton pump inhibitor	30 (19.7)	38 (21.1)	0.757
H2‐receptor antagonist	3 (2.0)	7 (3.9)	0.310
**Laboratory tests (mean ± SD)**			
White blood cells (cells/μL)	9880 ± 5520	9520 ± 3883	0.501
Hemoglobin (g/L)	8.4 ± 3.2	9.0 ± 2.9	0.048
Hematocrit (%)	25.4 ± 9.4	27.3 ± 8.8	0.065
Platelets (10⁴/μL)	22.3 ± 9.8	22.0 ± 9.4	0.739
PT‐INR	1.4 ± 1.4	1.1 ± 0.4	0.013
Albumin (g/dL)	3.2 ± 0.7	3.3 ± 0.6	0.555
Creatinine (mg/dL)	1.7 ± 1.9	1.4 ± 1.4	0.086
Blood urea nitrogen (mg/dL)	43.0 ± 34.8	40.9 ± 29.5	0.557
C‐reactive protein (mg/dL)	1.5 ± 3.4	1.6 ± 3.5	0.796
Shock index ≥1, *n* (%)	43 (28.3)	39 (21.7)	0.163
Glasgow Blatchford Score (mean ± SD)	12.7 ± 3.6	12.9 ± 3.6	0.280
AIMS65 Score (mean ± SD)	1.4 ± 1.0	1.3 ± 0.8	0.605
Complete Rockall score (mean ± SD)	4.2 ± 2.0	4.1 ± 1.9	0.504
Omeprazole (20 mg bolus i.v.)	97 (63.8)	84 (46.7)	0.002
Units of red cells by transfusion (mean ± SD)	3.9 ± 3.8	3.2 ± 3.9	0.087

*Note*: Missing data values for PT‐INR were 2.7%. Missing data values for CRP were 1.2%. Omeprazole was administered before upper gastrointestinal endoscopy.

Abbreviations: COVID‐19, coronavirus disease 2019; DAPT, dual anti‐platelet therapy; i.v., intravenous injection; NSAIDs, non‐steroidal anti‐inflammatory drugs; SD, standard deviation.

### COVID‐19‐related examinations, timings of endoscopy, and endoscopic findings

The COVID‐19 test results and the time to endoscopy are summarized in Table 2. Only one patient tested positive for COVID‐19 during the study period. The percentage of endoscopists who wore full PPE during the COVID‐19 pandemic was 100%. The mean time to emergency upper GI endoscopy was significantly longer during the COVID‐19 pandemic than before the pandemic (11.7 vs. 6.1 h; *p* < 0.01). Urgent endoscopy was significantly lower during the COVID‐19 pandemic than before (57.2% vs. 77.2%; *p* < 0.01). The time slot during which endoscopy was performed did not differ between the study periods (*p* = 0.23).

**TABLE 2:** COVID‐19‐related examination and timing of endoscopy.

	During the COVID‐19 pandemic (*n* = 152)	Before the COVID‐19 pandemic (*n* = 180)	*p*‐value
**COVID‐19‐related examinations**			
Chest CT examination rate, *n* (%)	121 (79.6)	76 (42.2)	<0.001
COVID‐19 antigen testing/PCR testing, *n* (%)	111 (73.0)	–	–
COVID‐19‐positivity rate, *n* (%)	1 (0.7)	–	–
**Timing of endoscopy**			
Time from presentation to endoscopy (hours ± SD)	11.7 ± 15.5	6.1 ± 8.7	<0.001
Urgent endoscopy, *n* (%)	87 (57.2)	139 (77.2)	<0.001
Early endoscopy, *n* (%)	46 (30.3)	34 (18.9)	0.016
Endoscopy (after 24 h), *n* (%)	19 (12.5)	7 (3.8)	0.004
**Time of endoscopy, *n* (%)**			
Regular working hours (09:00–17:00 h)	113 (74.3)	123 (68.3)	0.229
Outside regular working hours (17:01–08:59 h)	39 (25.7)	57 (31.7)	‐

*Note*: Urgent endoscopy defined as an endoscopy performed within 6 h of presentation, Early endoscopy defined as an endoscopy performed within 6–24 h after the patient's arrival at the hospital. Endoscopy (after 24 h) defined as an endoscopy performed after 24 h after the patient's arrival at the hospital.

Abbreviations: COVID‐19, coronavirus disease 2019; CT, computed tomography; PCR, polymerase chain reaction; SD, standard deviation.

The endoscopic findings are summarized in Table 3. Peptic ulcer was the most common cause of bleeding in both periods, accounting for approximately half of the cases. During and before the COVID‐19 pandemic, the number of cases of peptic ulcer associated with *Helicobacter pylori* was 38 (25.0%) and 43 (23.9%), respectively (*p* = 0.81). The site and rate of active bleeding did not significantly differ between the two periods. During and before the COVID‐19 pandemic, the proportions of lower GI endoscopy procedures for GI bleeding of unidentified cases were 8/27 (29.6%) and 11/29 (37.9%) cases, respectively (*p* = 0.51). Small bowel endoscopy was not conducted in any patient during either period.

**TABLE 3:** Endoscopic findings.

	During the COVID‐19 pandemic (*n* = 152)	Before the COVID‐19 pandemic (*n* = 180)	*p*‐value
**Disease, *n* (%)**			
Gastric ulcer	52 (34.2)	65 (36.1)	0.718
Duodenal ulcer	20 (13.2)	19 (10.6)	0.463
Malignant neoplasm	14 (9.2)	11 (6.1)	0.286
GAVE	6 (3.9)	12 (6.7)	0.276
Mallory–Weiss syndrome	6 (3.9)	12 (6.7)	0.276
GERD	5 (3.3)	4 (2.2)	0.551
GIST	2 (1.3)	5 (2.8)	0.356
Others	20 (13.2)	23 (12.8)	0.918
Unidentified	27 (17.8)	29 (16.1)	0.689
**Location, *n* (%)**			
Esophagus	3 (2.0)	4 (2.2)	0.875
Esophagogastric junction	12 (7.9)	17 (9.4)	0.618
**Stomach**			
U	16 (10.5)	27 (15.0)	0.226
M	43 (28.3)	44 (24.4)	0.427
L	30 (19.7)	32 (17.8)	0.648
Duodenal bulb	17 (11.2)	21 (11.7)	0.890
Second part	6 (3.9)	6 (3.3)	0.765
**Forrest's classification**			
Ia	9 (5.9)	18 (10.0)	0.176
Ib	12 (7.9)	13 (7.2)	0.817
IIa	35 (23.0)	38 (21.1)	0.675
IIb	0 (0)	2 (1.1)	0.192
IIc	1 (0.7)	2 (1.1)	0.664
III	18 (11.8)	16 (8.9)	0.377

Abbreviations: COVID‐19, coronavirus disease 2019; GAVE, gastric antral vascular ectasia; GERD, gastroesophageal reflux disease; GIST, gastrointestinal stromal tumor.

### Clinical outcomes

The clinical outcomes are summarized in Table 4. Regarding the primary outcomes, both univariate and multivariate analyses revealed that mortality within 30 days (adjusted OR: 2.27, *p* = 0.26), rebleeding within 30 days (adjusted OR: 0.43, *p* = 0.17), need for IVR/surgery (adjusted OR: 1.79, *p* = 0.33), and composite outcome (adjusted OR: 0.98, *p* = 0.96) did not differ significantly between the periods. Regarding the secondary outcomes, both univariate and multivariate analyses revealed that procedures to establish endoscopic hemostasis (adjusted OR: 0.38, *p* < 0.01) and second‐look endoscopy (adjusted OR: 0.04, *p* < 0.01) were less likely to be performed during the COVID‐19 pandemic as compared with that before the pandemic. The need for blood transfusion (adjusted OR: 1.61, *p* = 0.06) and prolonged hospitalization (OR: 0.98, *p* = 0.93) did not differ significantly between both periods. The cause of death was exacerbation of the primary disease or pneumonia due to GI bleeding in all cases, and there was no hemorrhagic death in any case (Table S1).

**TABLE 4:** Clinical outcomes by univariate and multivariate regression analyses.

	During the COVID‐19 pandemic (*n* = 152)	Before the COVID‐19 pandemic (*n* = 180)	Unadjusted OR (95% CI)	*p*‐value	Adjusted OR (95% CI)	*p*‐value
**Primary outcomes**						
Mortality within 30 days, *n* (%)	6 (3.9)	3 (1.6)	2.42 (0.60–9.86)	0.216	2.27 (0.55–9.48)	0.260
Rebleeding within 30 days, *n* (%)	4 (2.6)	10 (5.6)	0.46 (0.14–1.50)	0.197	0.43 (0.13–1.44)	0.173
IVR/Surgery, *n* (%)	8 (5.3)	5 (2.8)	1.94 (0.62–6.07)	0.252	1.79 (0.56–5.69)	0.325
Composite outcome, *n* (%)	15 (9.9)	17 (9.4)	1.05 (0.51–2.18)	0.896	0.98 (0.46–2.07)	0.956
**Secondary outcomes**						
Endoscopic hemostasis rate, *n* (%)	67 (44.1)	105 (58.3)	0.56 (0.36–0.87)	0.010	0.38 (0.23–0.64)	<0.001
Second‐look endoscopy, *n* (%)	66 (43.4)	105 (58.3)	0.55 (0.35–0.85)	0.007	0.04 (0.25–0.67)	<0.001
Blood transfusion units ≥2, *n* (%)	107 (70.4)	108 (60.0)	1.59 (1.00–2.51)	0.049	1.61 (0.98–2.66)	0.061
Prolonged hospitalization (≥14 days), *n* (%)	46 (30.3)	53 (29.4)	1.03 (0.65–1.67)	0.871	0.98 (0.59–1.61)	0.933

*Note*: Adjusted for sex, an AIMS65 score ≥2, and a complete Rockall score ≥5.

Abbreviations: COVID‐19, coronavirus disease 2019; CI, confidence interval; IVR, interventional radiology; OR, odds ratio..

A subgroup analysis revealed that clinical outcomes in the high‐risk group did not differ significantly between both periods (Table 5).

**TABLE 5:** Univariate and multivariate regression analyses of the clinical outcomes in the high‐risk group.

	During the COVID‐19 pandemic (*n* = 55)	Before the COVID‐19 pandemic (*n* = 55)	Unadjusted OR (95% CI)	*p*‐value	Adjusted OR (95% CI)	*p‐*value
**Primary outcomes**						
Mortality within 30 days, *n* (%)	2 (3.6)	2 (3.6)	1.00 (0.14–7.36)	1.000	0.93 (0.12–7.20)	0.944
Rebleeding within 30 days, *n* (%)	3 (5.5)	6 (10.9)	0.47 (0.11–1.99)	0.306	0.49 (0.11–2.10)	0.335
IVR/surgery *n* (%)	4 (7.3)	3 (5.5)	1.36 (0.29–6.38)	0.697	1.33 (0.28–6.42)	0.722
Composite outcome *n* (%)	8 (14.5)	10 (18.2)	0.77 (0.28–2.11)	0.606	0.76 (0.27–2.13)	0.602
**Secondary outcomes**						
Endoscopic hemostasis rate, *n* (%)	33 (60.0)	38 (69.1)	0.67 (0.31–1.47)	0.320	0.52 (0.21–1.26)	0.148
Second‐look endoscopy, *n* (%)	33 (60.0)	38 (69.1)	0.67 (0.31–1.47)	0.320	0.62 (0.27–1.41)	0.256
Blood transfusion units ≥2 *n* (%)	50 (90.9)	48 (87.3)	1.46 (0.43–4.91)	0.542	1.81 (0.48–6.93)	0.383
Prolonged hospitalization (≥14 days), *n* (%)	23 (41.8)	17 (30.9)	1.61 (0.73–3.52)	0.236	1.65 (0.72–3.76)	0.234

*Note*: Patients with GBS ≥12 points comprised the high‐risk group. Adjusted for sex, an AIMS65 score ≥2, and a complete Rockall score ≥5.

Abbreviations: COVID‐19, coronavirus disease 2019; CI, confidence interval; IVR, interventional radiology; OR, odds ratio.

The rates of endoscopic hemostasis in the groups receiving and not receiving omeprazole before endoscopy are shown in Table 6. The rate of endoscopic hemostasis was significantly lower in patients who received omeprazole before endoscopy compared with those who did not receive omeprazole (*p* = 0.03), other than peptic ulcer. However, the rate of endoscopic hemostasis did not differ between the two groups in the overall (*p* = 0.41) or peptic ulcer cohort (*p* = 0.88).

**TABLE 6:** Receiving versus not receiving proton pump inhibitor (PPI) before endoscopy.

	PPI (+)	PPI (−)	*p*‐value
**Over all**	***n* = 181**	***n* = 151**	
**Characteristics**			
Sex (male/female), *n* (%)	128 (70.7)/53 (29.3)	100 (66.2)/51 (33.8)	0.379
Age (mean ± SD), years	73.6 ± 14.4	71.2 ± 16.8	0.160
Charlson Comorbidity Index (mean ± SD)	0.8 ± 1.3	0.8 ± 2.2	0.831
Glasgow Blatchford Score (mean ± SD)	10.1 ± 3.4	9.2 ± 3.7	0.033
AIMS65 Score (mean ± SD)	1.4 ± 0.9	1.3 ± 0.8	0.125
**Outcomes**			
Endoscopic hemostasis rate, *n* (%)	90 (49.7)	82 (54.3)	0.405

**Peptic ulcer**	***n* = 87**	***n* = 69**	
**Characteristics**			
Sex (male/female), *n* (%)	71 (81.6)/16 (18.4)	46 (66.7)/23 (33.3)	0.032
Age (mean ± SD), years	71.5 ± 14.8	73.2 ± 15.8	0.500
Charlson Comorbidity Index (mean ± SD)	0.5 ± 0.9	0.8 ± 2.3	0.311
Glasgow Blatchford Score (mean ± SD)	10.8 ± 2.9	10.1 ± 3.2	0.153
AIMS65 Score (mean ± SD)	1.5 ± 1.0	1.3 ± 0.9	0.245
**Outcomes**			
Endoscopic hemostasis rate, *n* (%)	64 (73.6)	50 (72.5)	0.878

**Other than peptic ulcer**	***n* = 66**	***n* = 54**	
**Characteristics**			
Sex (male/female), *n* (%)	42 (63.6)/24 (36.4)	37 (68.5)/17 (31.5)	0.575
Age (mean ± SD), years	76.7 ± 13.6	69.3 ± 16.5	0.009
Charlson Comorbidity Index (mean ± SD)	1.2 ± 1.6	1.1 ± 2.4	0.802
Glasgow Blatchford Score (mean ± SD)	9.8 ± 3.8	9.2 ± 3.8	0.372
AIMS65 Score (mean ± SD)	1.4 ± 0.8	1.3 ± 0.8	0.588
**Outcomes**			
Endoscopic hemostasis rate, *n* (%)	26 (39.4)	32 (59.3)	0.030

*Note*: Other than peptic ulcer, excluding those with unknown source of bleeding.

Abbreviation: SD, standard deviation.

## DISCUSSION

This study focused on the endoscopic management and clinical outcomes of patients with NVUGIB during the COVID‐19 pandemic. First, 180 and 152 emergency upper GI endoscopies were performed before and during the COVID‐19 pandemic, respectively (Table 1); the number of emergency upper GI endoscopies did not decrease markedly during the COVID‐19 pandemic. Second, although the time to emergency endoscopy was significantly longer during the COVID‐19 pandemic (Table 2), it did not affect the rates of mortality, rebleeding, IVR/surgery requirement, and composite outcome, even in severe cases (Tables 4 and 5). Third, the rates of endoscopic hemostasis procedures and second‐look endoscopies decreased significantly during the COVID‐19 pandemic (Table 4). Our results indicate that the time delay in endoscopic management during the pandemic period did not affect the mortality and rebleeding of patients with NVUGIB. These results may help physicians involved in the endoscopic management of these patients during the next wave of the COVID‐19 pandemic, as well as during the next infectious disease pandemic (should it occur).

Previous studies have revealed a 30%–80% decrease in the number of emergency upper GI endoscopies performed during the COVID‐19 pandemic in Western countries.^3^, ^26^ However, in this study, we did not observe a marked decrease in the number of emergency upper GI endoscopies performed during the pandemic. One reason for this may be that our hospitals were equipped to provide PCR test results and thoracic CT scans immediately for patients with COVID‐19. Furthermore, adequate infection control, including the use of droplet‐preventing masks, appropriate ventilation, and complete PPE, enabled us to undertake emergency endoscopies during the COVID‐19 pandemic.

Important clinical outcomes, such as mortality, rebleeding, IVR/surgery requirement, and composite outcome, did not differ significantly between before and during the COVID‐19 pandemic in our study, which is consistent with previous reports.^9^, ^10^, ^11^ Khan et al., in a study on 1192 patients with upper GI bleeding, found no significant differences between before and during the pandemic in the rates of mortality (4.2% vs. 3.9%) and rebleeding (10.7% vs. 10.0%).^9^ Moreover, in a study on 211 patients with upper GI bleeding, Kim et al. found no significant difference in the mortality rate between before and during the pandemic (8.9% vs. 10.2%).^10^

Notably, we found that the time to emergency upper GI endoscopy was significantly longer during the COVID‐19 pandemic (11.7 h) than before the pandemic (6.1 h). There are two possible reasons for this. First, it took time to perform COVID‐19‐related tests (e.g., PCR and chest CT) to prevent exposing the endoscopists and medical staff to the infection. Second, medical and human resources were directed toward the management of respiratory diseases, diminishing the priority allocated to endoscopy centers.^27^ The rate of endoscopic hemostasis decreased significantly during the COVID‐19 pandemic in this study. A review of 2223 cases of upper GI bleeding^28^ and a randomized control trial of patients with upper GI bleeding^7^, ^24^ concluded with a moderate level of evidence that PPI administration before endoscopy reduces the requirement of endoscopic treatment. The exposed vessels might have disappeared since there was sufficient time for PPI administration prior to endoscopy, reducing the need for endoscopic treatment.^7^, ^29^ In fact, in our study, the rate of PPI administration before upper GI endoscopy was significantly higher during the pandemic period. However, in our study, PPI administration before endoscopy significantly reduced the rate of endoscopic hemostasis among patients with diseases other than peptic ulcers but not among patients with peptic ulcers. A possible reason for this is the difference in PPI doses between Japan and Western countries. In Western countries, a high‐dose intravenous infusion of 8 mg every hour after a bolus dose of 80 mg of PPI is recommended.^30^ However, such dose is not covered by national insurance in Japan and omeprazole should only be used at a maximum dose of 40 mg per day. Second‐look endoscopies also decreased significantly during the pandemic, likely due to the reduction of unnecessary endoscopy procedures according to the guidelines.^4^, ^5^

One COVID‐19‐positive patient was identified in this study; because his general condition and vital signs were stable, he received conservative treatment with PPI and supplemental fluids. After a 1‐week isolation, the patient underwent endoscopy with minimal staff in full PPE; thereafter, the patient was discharged without any issues. However, not all patients will have such an uneventful course.^31^ In COVID‐19‐positive patients with NVUGIB requiring emergency treatment, IVR without aerosol generation may be an alternative to reduce the infection risk to the medical staff.^32^

This study has some limitations. First, it was a retrospective study of a small number of patients. Second, it was conducted at tertiary centers capable of conducting emergency endoscopies and CT around the clock. Third, the decision to perform endoscopic hemostasis procedures and the choice of the hemostatic technique were left at the discretion of the endoscopists. Fourth, the possibility of a selection bias exists. During the COVID‐19 period, only patients with a high need for emergency endoscopy may have been selected as eligible patients. There was no statistically significant difference in the GBS score, AIMS65 score, and complete Rockall score that predicted severity between the two periods. However, in the laboratory test at arrival hospitals, the hemoglobin level was significantly lower (*p* = 0.048) and PT‐INR level was significantly higher (*p* = 0.01) during the COVID‐19 period. Finally, the use of standard precautions before the COVID‐19 pandemic was left to the endoscopist's discretion, and the compliance rate could not be investigated. Although there are these limitations, the strengths of the present study were the detailed investigation of endoscopic management, such as the percentages of endoscopic diagnosis and hemostasis related to the outcome, pre‐endoscopic PPI utilization, and examination time from the hospital visit to endoscopy.

In conclusion, although the time to emergency upper GI endoscopy was significantly longer during the pandemic, it had no effect on the rates of mortality, rebleeding, IVR/surgery requirement, and composite outcome. These results may help physicians involved in the endoscopic management of patients with NVUGIB during the next wave of the COVID‐19 pandemic and the next pandemic of a respiratory infectious disease.

## CONFLICT OF INTEREST STATEMENT

Authors declare no conflicts of interest for this article. This work was partially supported by the Japanese Foundation for Research and the Promotion of Endoscopy Grant. The funders played no role in the study design, data analysis, and decision to publish the manuscript.

## ETHICS STATEMENT

This study was conducted in accordance with the Declaration of Helsinki. The study protocol was approved by the St. Marianna University School of Medicine ethics committee. Informed consent was obtained via an opt‐out manner on the intuitional website.

## Supporting information

Table S1 Patient's cause of death
