# Supplementary material for: Impact of the COVID‐19 pandemic on the time to emergency endoscopy and clinical outcomes in patients with upper gastrointestinal bleeding
Source: DEN Open. 2023 Nov 10;4(1):e310. doi: 10.1002/deo2.310 (PMC10638502; doi:10.1002/deo2.310)
Supplement: Supplementary file 1 — Table S1 Patient's cause of death [file DEO2-4-e310-s001.docx]

| **Supplementary Table 1.** Patient's cause of death | | | | | |
| --- | --- | --- | --- | --- | --- |
| **Patient** | **Age** | **Sex** | **Cause of bleeding** | **Comorbidities** | **Cause of death** |
| 1 | 50 | Male | Others | Cirrhosis of liver | Liver failure |
| 2 | 51 | Male | Unidentified | Cirrhosis of liver | Liver failure |
| 3 | 77 | Male | Gastric cancer | Gastric cancer | Sepsis |
| 4 | 77 | Male | Gastric cancer | Cerebrovascular disease, Chronic kidney disease | Aspiration pneumonia |
| 5 | 81 | Male | Gastric antral vascular ectasia | Hepatic cell cancer | Aspiration pneumonia |
| 6 | 84 | Male | Unidentified | Chronic kidney disease | Pneumonia |
| 7 | 86 | Male | Gastric ulcer | Cerebrovascular disease, Diabetes | Aspiration pneumonia |
| 8 | 91 | Male | Invasive of carcinoma in the stomach from other organ | Cerebrovascular disease | Perihilar cholangiocarcinoma |
| 9 | 94 | Female | Mallory–weiss syndrome | Cardiovascular disease, Chronic kidney disease | Aspiration pneumonia |
|  | | | | | |
